# Delivering a RAS protease halts tumor growth

**DOI:** 10.18632/oncotarget.27714

**Published:** 2020-09-01

**Authors:** Vania Vidimar, Roman A. Melnyk, Karla J.F. Satchell

**Keywords:** RAS, RRSP, chimeric toxins, cancer, xenografts

***Comment on:*** *Vidimar V, et al. A novel engineered chimeric toxin that cleaves activated mutant and wild-type RAS inhibits tumor growth. Proc Natl Acad Sci U S A. 2020; 117:16938–16948. https://doi.org/10.1073/pnas.2000312117. [PubMed]*

More than one-third of all human cancers are driven by mutations in *RAS* genes, which were among the first oncogenes discovered about four decades ago. *RAS* genes (*H-*, *N-*, and *KRAS*) encode small membrane-associated GTPases that act as central molecular switches in signaling networks involved in differentiation, proliferation and survival. Because of their role and impact on cancer, RAS proteins are among the most coveted pharmacological targets for cancer therapy. However, the high affinity for GTP and lack of accessible drug-binding pockets have made anti-RAS drug design difficult and RAS proteins have been historically considered undruggable. Currently, there are no FDA-approved therapies against RAS in the clinic [1]. Yet recently, there has been a renewed interest in tackling RAS directly, with at least four inhibitors that target KRAS G12C under evaluation in Phase I or Phase II clinical trials [2]. However, clinical benefits with these molecules are restricted to patients with KRAS G12C-driven tumors, which account for < 14% of all KRAS cancers. Thus, additional efforts are needed to identify effective inhibitors that target the major RAS oncoproteins as well as aberrantly overactivated wild-type RAS proteins so that a larger pool of patients with cancer, and particularly RAS-driven cancers, can benefit.

Bacterial toxins are known to exhibit high specificity for their host intracellular targets, which are primarily proteins and central regulators of signal transduction such as small GTPases. Indeed, the bacterial RAS-RAP1 specific endopeptidase (RRSP) specifically cleaves both active and inactive RAS and RAP1, leaving other closely related members of the RAS family unaffected [3–5]. In addition, polymorphic toxins such as diphtheria toxin can be disarmed of the catalytically active domain and the remaining domains employed as transduction platforms to deliver bioactive proteins via a receptor-binding mechanism [6]. This receptor-mediated delivery is highly important because the principle underlying targeted cancer therapy takes advantage of cancer-specific cell surface molecules as recognition sites for selective targeting and elimination of neoplastic cells, while sparing healthy cells. In our recent paper published in the *Proc. Natl. Acad. Sci. USA*, we described the development of a biologic chimeric toxin comprising RRSP fused to the translocation and receptor-binding domains of diphtheria toxin (DT_B_) [7]. We showed that DT_B_ specifically and efficiently delivers RRSP to cancer cell lines bearing the diphtheria toxin receptor HB-EGF and then abrogates cell viability and proliferation at concentrations as low as one picomolar. Assessment of the efficacy of this toxin to inhibit cell growth across the entire NCI-60 panel revealed that nearly all cancer lines tested were susceptible to RRSP-DT_B_. Those classified as most susceptible to RRSP-DT_B_ commonly have genomic abnormalities in *RAS* genes.

**Figure 1:**
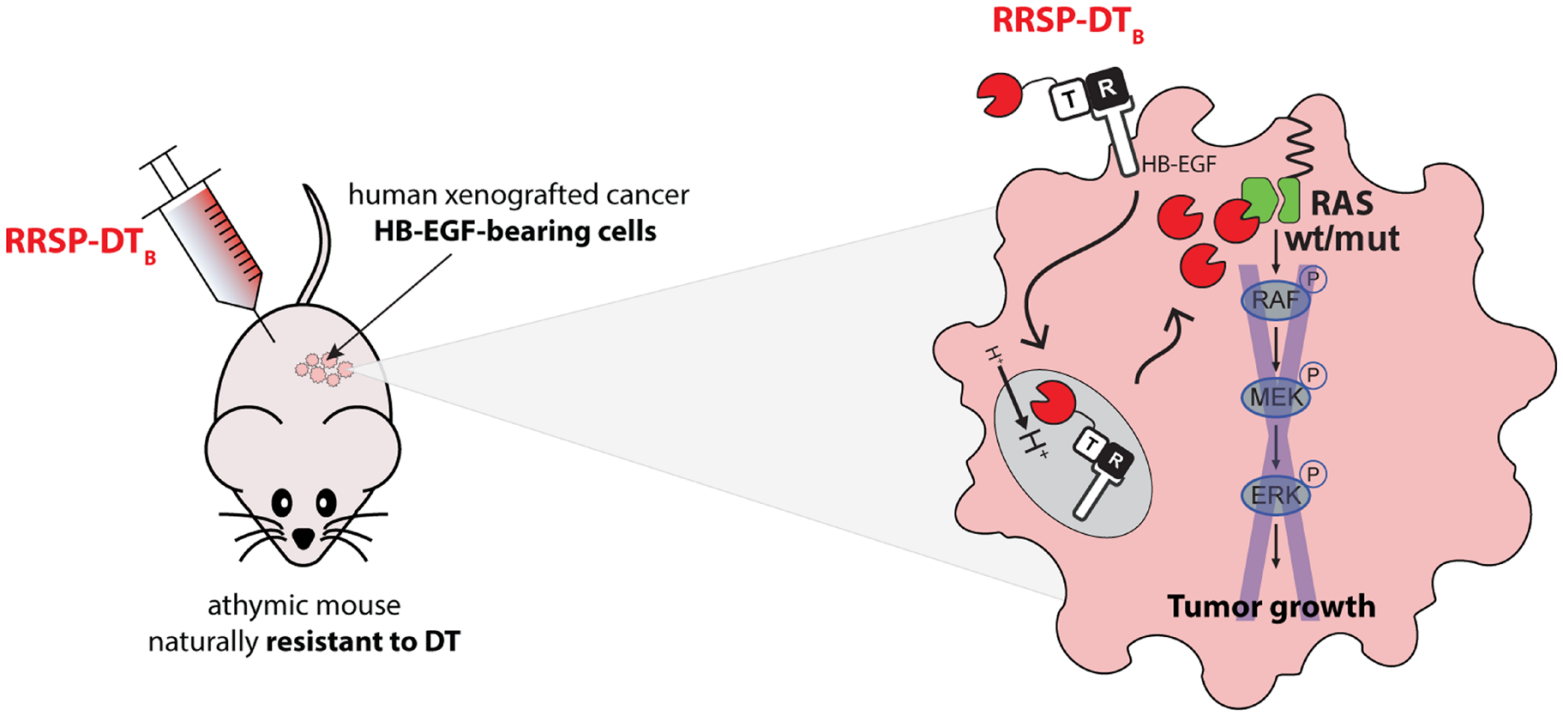
Schematic illustration depicting simplified delivery of RRSP to xenografted HB-EGF-bearing cancer cells in mice via DT_B_. The DT_B_ receptor R domain recognizes HB-EGF on the surface of xenografted human cancer cells in athymic mice and the all complex is internalized into intracellular endosomes. Following acidification of the endosomal compartments, the translocation T domain of DT_B_ allows release of RRSP into the cytosol, where RRSP engages and cleaves RAS at the membrane, thereby ablating downstream RAS signaling and ultimately halting tumor growth.

Because mice are naturally resistant to diphtheria toxin, its administration does not cause toxicity and thus the DT_B_ portion of the chimeric toxin functions as a human cancer cell specific targeting strategy in mice. We showed that athymic nude mice did not experience toxicity at the doses of RRSP-DT_B_ employed and RRSP was released via DT_B_ only to the xenografted HB-EGF-expressing human cancer cells, leading to specific RAS target engagement and reduced growth in tumors driven by either wild-type or mutant RAS (Figure 1). Moreover, due to the modularity of DT_B_, improved tumor-selective targeting can be achieved by replacing the receptor-binding domain of DT_B_ with other binding moieties, such as interleukin-2 (IL-2), so that cancer cells overexpressing the IL-2 receptor (IL-2R) can be more efficiently targeted by RRSP-DTT-IL2.

This work established the potential for use of RRSP as a therapeutic to reduce tumorigenesis and for the translocation component of diphtheria toxin to transfer cargoes into cancer cells *in vivo*. The pairing of these toxin components in RRSP-DT_B_ shows efficacy to treat *in vivo* models of RAS-driven tumors. For therapeutic purposes, development of a human-tailored, targeted delivery of RRSP to cancer cells is planned in order to limit any on-target off-tumor toxicity. For the DT_B_-based platform to be used as the ultimate delivery strategy, common DT_B_ epitopes need to be modified in order to circumvent immunogenicity, while retaining translocation functions. In addition, DT_B_ can be modified in a cassette fashion to target cancer specific receptors. Alternative possibilities include packaging RRSP or RRSP-DT_B_ into ligand-targeted carriers for assisted delivery of RRSP to cancer cells that selectively express or overexpress specific receptors. Our work provides evidence of the *in vivo* anticancer effect of a bacterial toxin with built-in RAS proteolytically activity. We envision that further efforts towards finding a delivery system for RRSP with more translatable impact may take anti-RAS therapy to the next level.
